# Redox and proteolytic regulation of cardiomyocyte β_1_-adrenergic receptors – a novel paradigm for the regulation of catecholamine responsiveness in the heart

**DOI:** 10.3389/fimmu.2023.1306467

**Published:** 2023-12-04

**Authors:** Susan F. Steinberg

**Affiliations:** Department of Molecular Pharmacology and Therapeutics, Columbia University, New York, NY, United States

**Keywords:** β1-adrenergic receptors, elastase, oxidative stress, cardiomyocytes, proteolysis

## Abstract

Conventional models view β_1_-adrenergic receptors (β_1_ARs) as full-length proteins that activate signaling pathways that influence contractile function and ventricular remodeling - and are susceptible to agonist-dependent desensitization. This perspective summarizes recent studies from my laboratory showing that post-translational processing of the β_1_-adrenergic receptor N-terminus results in the accumulation of both full-length and N-terminally truncated forms of the β_1_AR that differ in their signaling properties. We also implicate oxidative stress and β_1_AR cleavage by elastase as two novel mechanisms that would (in the setting of cardiac injury or inflammation) lead to altered or decreased β_1_AR responsiveness.

## 1 Introduction

**Figure 1:**
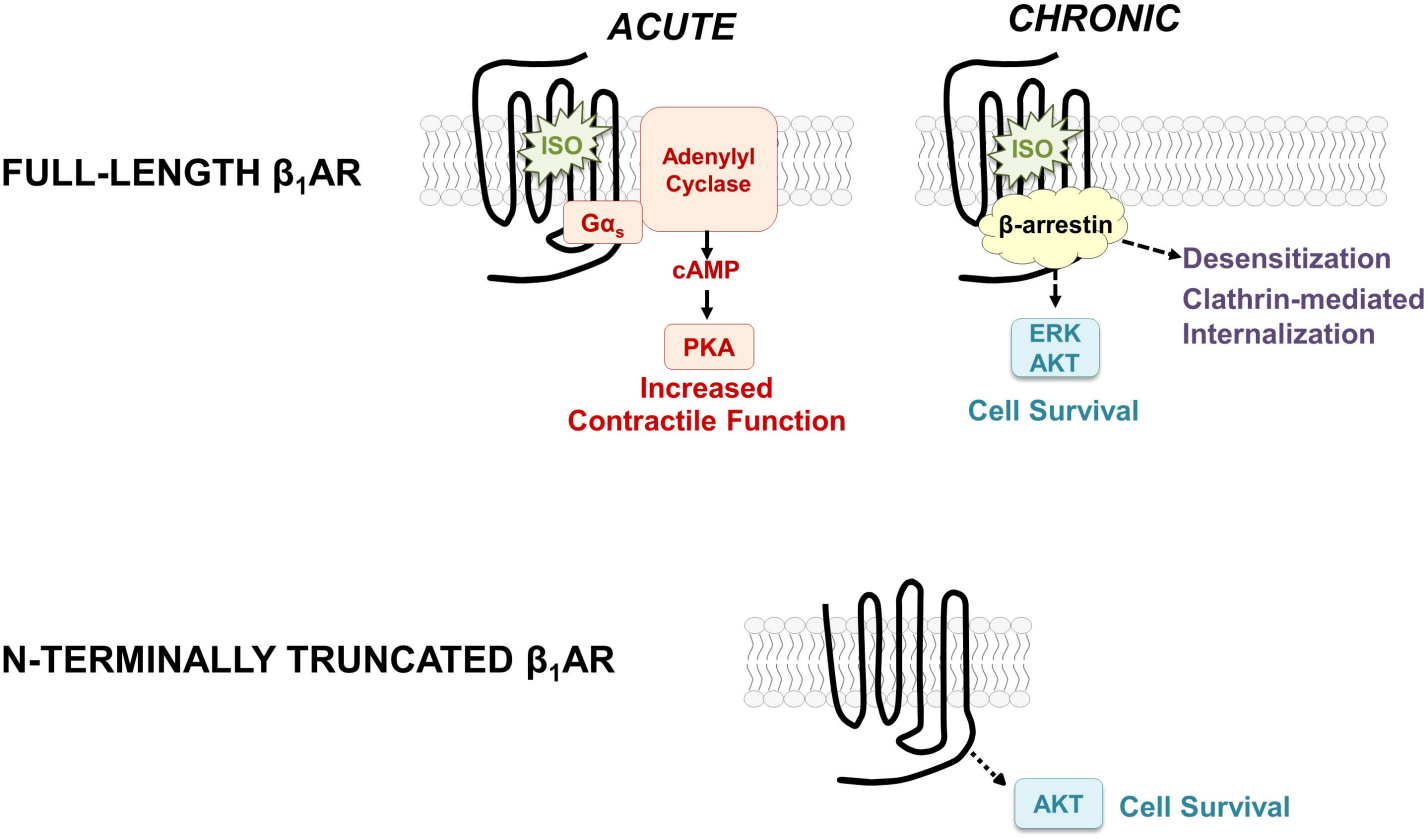
Schematic showing the classical paradigm for signaling by full-length β_1_ARs (top) and the distinct signaling properties of the N-terminally truncated form of the β_1_AR (bottom).

β-Adrenergic receptors (βARs) are among the most intensively studied members of the G protein-coupled receptor superfamily primarily because they control physiologic mechanisms that impact on the pathogenesis of cardiovascular disease; they are clinically important targets for drug discovery. Conventional models hold that βARs function to rapidly adjust cardiac output by recruiting a Gs-adenylyl cyclase pathway that leads to the accumulation of cAMP, activation of protein kinase A, and phosphorylation of membrane and sarcomeric proteins involved in excitation-contraction coupling (**Figure 1**). βAR-driven inotropic and chronotropic responses provide hemodynamic support in the setting of acute heart failure. However, with chronic heart failure, agonist-occupied βARs are stabilized in an active conformation that is phosphorylated by G protein-coupled receptor kinase (GRK); GRK-phosphorylated receptors then recruit β-arrestin, an adapter protein that acts to both sterically interdict βAR-G protein interactions and scaffold binding partners that trigger a second wave of signaling to Gs-independent growth regulatory responses such as extracellular-signal regulated kinase (ERK) and AKT (1, 2). While these pathways generally have been implicated in cardioprotection, studies in cardiomyocytes also link chronic βAR activation the activation of proapoptotic pathways and a spectrum of changes (including cardiomyocyte hypertrophy and apoptosis, interstitial fibrosis, and contractile dysfunction) that contribute to the pathogenesis of heart failure (3, 4).

It is worth noting that conventional models describing the molecular basis for βAR signaling responses derive from literature heavily biased towards an analysis of the β_2_AR. While the literature has generally tended to assume that the signaling properties of β_1_ARs and β_2_ARs are similar (and that β_2_ARs can serve as meaningful surrogates for the β_1_AR subtype), this ignores a substantial body of evidence showing that β_1_ARs are relatively resistant to agonist-induced GRK-dependent phosphorylation, they engage β-arrestin only weakly (5), and they show little-to-no agonist-induced internalization (6, 7). These differences should not be surprising, since the 54% overall homology between β_1_AR and β_2_AR subtypes is largely confined to their transmembrane, ligand-binding pockets; their N- and C-termini are quite distinct (8). This perspective summarizes our recent studies focusing on the β_1_AR N-terminus, the relatively short/unstructured extracellular portion of the receptor that differs in length, sequence, and post-translational processing from the β_2_AR N-terminus. Our studies characterize an O-glycan regulated N-terminal cleavage mechanism that is specific for the β_1_AR (does not apply to the β_2_AR) that results in the generation of N-terminally truncated β_1_ARs with signaling properties that differ from that described for full-length β_1_ARs (9, 10). Our results force a reexamination of current concepts regarding the molecular basis for β_1_AR-dependent signaling responses, since conventional models have been derived from literature predicated on the assumption that β_1_ARs signal exclusively as full-length receptors. This perspective focuses on the novel notion β_1_AR cleavage results in the generation of distinct β_1_AR species that differ in their signaling properties.

## 2 Post-translational processing of the β_1_AR

### 2.1 β_1_AR O-glycosylation

**Figure 2:**
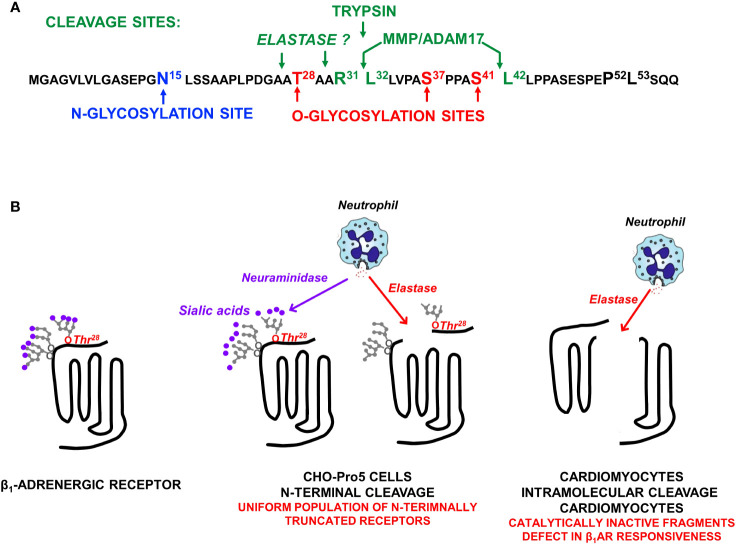
**(A)** The β_1_AR N-terminal sequence showing the single N-glycosylation site at N^15^, O-glycosylation sites at T^28^, S^37^, and S^41^, and the major ADAM17 cleavage site at R^31^↓L^32^ as well as a second ADAM17 cleavage site at S^41^↓L^42^ (that is exposed only when S^41^ O-glycosylation is prevented). **(B)** β_1_AR N-terminal cleavage by neutrophil elastase occurs only when the O-linked glycan at T^28^ is not capped by a terminal sialic acid; O-linked glycans at S^37^ and S^41^ do not impact on elastase-dependent β_1_AR N-terminal cleavage. Studies in neonatal rat ventricular cardiomyocytes expose an additional intramolecular cleavage site for elastase (that is not dependent on N-terminal glycan desialylation) that disrupts catecholamine responsiveness.

β_1_ARs and β_2_ARs both contain sites for N-glycosylation, but β_1_ARs uniquely serve as targets for O-glycosylation and proteolytic cleavage (**Figure 2A**). In way of background, N-glycosylation results from the *en bloc* transfer of a preformed complex glycan structure to an asparagine residue in a N-x-S/T consensus motif, with the glycan then further trimmed/modified to yield higher-order glycan structures. In contrast, O-glycosylation is a non-template driven post-translational modification that is initiated by the enzymatic transfer of a single α-GalNAc to an acceptor serine or threonine residue (a reaction catalyzed by a member of the multi-gene family of polypeptide GalNAc-transferases that differ in their tissue distribution and substrate specificities), followed by the step-wise enzymatic transfer of additional sugars (galactose, GlcNac, fucose, etc.) to yield a spectrum of complex higher-order linear and/or branched glycan structures (11). This mechanism allows for a high level of structural diversity/microheterogeneity, even at single sites within a given protein. Evidence that clusters of O-glycans can serve as barriers to prevent protein cleavage by proteases (11) provided the rationale to examine whether O-glycosylation play a role in the maturational processing of β_1_ARs.

We used a range of biochemical and mutagenesis approaches [described in detail in previous publications (9, 10)] to map the major β_1_AR N-terminal O-linked glycosylation sites to Ser^37^ and Ser^41^ and show that O-glycan structures are heavily sialylated (a feature that will become important in the section that follows). We showed that O-glycosylation is required for full-length β_1_AR expression; β_1_ARs accumulate as N-terminally truncated species under conditions that prevent β_1_AR O-glycosylation [suggesting that mucin-like O-glycans at the N-terminus act as barriers to prevent protease cleavage at adjacent sites (9)]. We also established the importance of this N-terminal processing mechanism, showing that N-terminal truncation alters the β_1_AR’s signaling bias between cAMP/PKA vs. ERK pathways and that N-terminally truncated β_1_ARs acquire a unique function to constitutively activate AKT and protect against doxorubicin-induced cardiomyocyte apoptosis [i.e., the β_1_AR acquires a cardioprotective phenotype as a result of N-terminal truncation, **Figure 1**, *bottom* (9, 10)]. These studies implicate the β_1_AR N-terminus as a heretofore-unrecognized structural determinant of β_1_AR responsiveness.

It is worth noting that the O-glycan-regulated N-terminal cleavage mechanism identified in our studies provides the first credible explanation for the molecular heterogeneity observed for native β_1_AR in various cardiac preparations. Our findings also emphasize that immunblotting or immunohistochemistry studies that rely on antibodies to N-terminal epitope tags to track β_1_AR expression and/or localization should be interpreted with caution, since these techniques do not capture N-terminally truncated forms of the β_1_AR.

### 2.2 β_1_AR N-terminal cleavage

We used mutagenesis and pharmacologic strategies [described in (9, 10)] to map the major O-glycan-regulated β_1_AR N-terminal cleavage site to R^31^↓L^32^ and show that cleavage at this site is attributable to the cellular actions of a disintegrin and metalloproteinase 17 [ADAM17; **Figure 2A** (9, 10)]. We also identified a secondary ADAM17-dependent N-terminal cleavage at S^41^↓L^42^ that is specifically inhibited by the O-glycan modification at S^41^ (10).

The observation that the β_1_AR N-terminus can serve as a target for proteolytic cleavage by ADAM17 raised the obvious question of whether full-length β_1_ARs on the cell surface are cleaved by other proteases. Since the R^31^↓L^32^ cleavage site conforms to a consensus trypsin cleavage motif, we examined whether β_1_ARs are cleaved by trypsin. We showed that trypsin cleaves full-length β_1_ARs and that cleavage is specifically at the N-terminal R^31^↓L^32^ cleavage site (**Figure 2A** (12),). This mechanism should interest laboratories that interrogate β_1_AR signaling in isolated adult cardiomyocytes – cells typically extracted from intact ventricular using trypsin digests (13). The *in vivo* significance of cardiomyocyte β_1_AR cleavage by trypsin (a digestive enzyme found in the gastrointestinal tract) is dubious.

Therefore, we turned our attention to other, more pathophysiologically relevant proteases. Preliminary studies performed in CHO-Pro5 cells failed to identify significant levels of β_1_AR cleavage by various proteases. Since terminal charged sialic acid residues have been implicated as regulators of glycoprotein cleavage (14–16) and O-glycan attachments on the β_1_AR N-terminus are heavily sialylated (9), we repeated the protease screens in cells treated with neuraminidase (an enzyme that removes terminal sialic acid residues). These studies exposed an action of elastase to cleave full-length (but not N-terminally truncated) β_1_ARs under conditions that disrupt glycoprotein sialylation [**Figure 2B** (17)]. We then used a mutagenesis strategy to identify the sialylated O-glycan attachment that prevents elastase cleavage. Preliminary studies effectively ruled out roles for previously identified N- or O-glycosylation sites at N^15^, S^37^, or S^41^. Therefore, we considered a possible role for T^28^, a residue previously reported to be O-glycosylated in the context of reductionist *in vitro* assays (18). These studies showed that a sialylated O-glycan at T^28^ plays only a minor role in the maturational processing of β_1_ARs to full-length receptors, but it fully protects β_1_ARs from elastase-dependent cleavage (17). This novel mechanism for β_1_AR regulation is predicted to have pathophysiologic importance, given that neuraminidase is released along with elastase by activated neutrophils at sites of inflammation or injury (**Figure 2**).

We then used a mutagenesis strategy in an attempt to map the elastase cleavage site. Our studies excluded possible roles for previously identified MMP/ADAM17-sensitive sites at R^31^↓L^32^ or S^41^↓L^42^ indicating that elastase cleavage must be at another site on the β_1_AR N-terminus (17). In this regard, it is intriguing to note that T^28^ is strategically positioned adjacent to an elastase consensus cleavage motif (i.e., elastase typically cleaves scissile bonds C-terminal to small residues such as Ala, Gly, or Val; **Figure 2A**).

An elastase-dependent cleavage mechanism restricted to the β_1_AR N-terminus would generate a uniform population of signaling-competent N-terminally truncated receptors – a molecular form of the β_1_AR that constitutively couples to the cardioprotective AKT pathway. Hence, this type of proteolytic cleavage mechanism is predicted to afford survival advantage in the setting of heart failure.

### 2.3 β_1_AR cleavage at an intramolecular site

The actions of elastase also were examined in the more physiologically relevant cardiomyocyte context. Here, elastase treatment (even under conditions that do not disrupt protein O-glycosylation or sialylation) leads to a decrease in the abundance of the β_1_AR in association with the accumulation of ~40 kD N-terminal and ~25 kD C-terminal fragments, consistent with an intramolecular cleavage in extracellular loop 2 (**Figure 2B** (17),). The additional observation that elastase treatment results in a pronounced defect in isoproterenol-dependent (but not basal or forskolin-dependent) cAMP accumulation (17) supports the conclusion that the β_1_AR fragments that accumulate in elastase-treated cardiomyocytes are signaling-incompetent.

Our studies linking elastase treatment to an intramolecular cleavage that disrupts β_1_AR responsiveness were performed in neonatal cardiomyocyte cultures. It is important to note that differences in β_1_AR glycosylation or trafficking patterns between neonatal and adult cardiomyocytes or in cardiomyocytes that have been induced to hypertrophy could in theory lead to differences in β_1_AR protease-sensitivity. Hence, the functional consequences of β_1_AR cleavage would depend upon whether cleavage is restricted to the N-terminus (resulting in a uniform population of cardioprotective β_1_ARs) or whether cleavage is at an intramolecular site that disrupts catecholamine responsiveness. Mechanisms that fine-tune β_1_AR protease-sensitivity are the subject of ongoing studies.

### 2.4 β_1_AR regulation by oxidative stress

There is considerable evidence that chronic heart failure (which is associated with elevated catecholamine levels) leads to a loss of cardiac reserve due to decreased expression and signaling by βARs. This loss of catecholamine responsiveness traditionally has been attributed to homologous desensitization and/or βAR down-regulation. However, the notion that a single mechanism underlies the heart failure-induced defects in signaling by both the β_1_AR (the predominant βAR subtype and principal driver of catecholamine-driven sympathetic responses in the healthy heart) and the β_2_AR subtype is difficult to reconcile with clinical studies showing that β_1_ARs and β_2_ARs are regulated differently in heart failure; heart failure leads to a selective downregulation of the β_1_AR subtype that is not accompanied by a commensurate loss of β_2_ARs. This formulation also is at odds with cell-based studies showing that β_2_ARs undergo agonist-dependent desensitization/down-regulation, but β_1_ARs are relatively resistant agonist-induced desensitization/internalization (6, 19). In this regard, it is worth noting that much like the N-termini, β_1_AR and β_1_AR C-termini and intracellular loops show little sequence homology; sites on the β_2_AR that serve as substrates for GRK phosphorylation and/or docking sites for β-arrestin are not conserved in the β_1_AR subtype. This raises a fundamental question as to the mechanism underlying the defect in β_1_AR responsiveness in heart failure. Our recent studies address this conundrum by showing that oxidative stress (a stimulus that contributes to the pathogenesis of heart failure and various other cardiomyopathic syndromes) decreases β_1_AR expression and isoproterenol responsiveness in cardiomyocytes; oxidative stress does not lead to changes in the expression of the β_2_AR subtype (20). Hence, these studies implicate oxidative stress as a mechanism that selectively decreases β_1_AR (but not β_2_AR) expression that would underly the decreased cardiac catecholamine responsiveness that is a hallmark of heart failure.

## 3 Discussion

Studies in model cell types have traditionally ignored possible differences in the biological controls and cellular actions of β_1_ARs vs. β_2_ARs. However, studies in genetic models of receptor overexpression provide compelling evidence that the deleterious effects of chronic sympathetic overdrive that contribute to the pathogenesis of cardiac hypertrophy and heart failure can be attributed to the cardiac actions of the β_1_AR subtype. These studies show that moderate levels of transgenic β_1_AR overexpression leads to maladaptive cardiac remodeling and heart failure (21, 22) whereas even high levels of transgenic β_2_AR overexpression are relatively well tolerated (23–25). Our studies add an additional dimension to the analysis by showing that the β_1_AR subtype accumulates as both full-length and N-terminally truncated forms and that these distinct molecular forms of the β_1_AR display importance differences in their coupling to pro- vs. anti-apoptotic signaling pathways. The notion that the β_1_AR N-terminus functions as a novel molecular determinant of β_1_AR signaling responses suggests that therapeutic strategies designed to influence β_1_AR N-terminal cleavage might be exploited for the treatment of heart failure. Our studies also implicate oxidative stress and proteolytic cleavage as two pathophysiologicaly relevant stimuli that act to either disrupt or alter catecholamine-driven β_1_AR growth and/or injury responses in the setting of heart failure, cardiac inflammation, or myocardial infarction-induced cardiac injury. Collectively, the novel signaling paradigms for cardiomyocyte β_1_ARs identified in our studies add a new dimension to our understanding of the evolution of heart failure and other cardiomyopathic disorders.

## Data availability statement

The original contributions presented in the study are included in the article/Supplementary Material. Further inquiries can be directed to the corresponding author.

## Ethics statement

The animal study was approved by Columbia University Institutional Animal Care and Use Committee. The study was conducted in accordance with the local legislation and institutional requirements.

## Author contributions

SS: Conceptualization, Funding acquisition, Supervision, Writing – review & editing.
